# From Historical Narratives to Circular Economy: De-Complexifying the “Desertification” Debate

**DOI:** 10.3390/ijerph17155398

**Published:** 2020-07-27

**Authors:** Rares Halbac-Cotoara-Zamfir, Andrea Colantoni, Enrico Maria Mosconi, Stefano Poponi, Simona Fortunati, Luca Salvati, Filippo Gambella

**Affiliations:** 1 Department of Overland Communication Ways, Foundation and Cadastral Survey, Politehnica University of Timisoara, 300224 Timisoara, Romania; raresh_81@yahoo.com; 2 Department of Agricultural and Forest Science, University of Tuscia, I-01100 Viterbo, Italy; 3 Department of Economics, Engeneering, Society and Business, University of Tuscia, I-01100 Viterbo, Italy; enrico.mosconi@unitus.it (E.M.M.); simonafortunati@unitus.it (S.F.); 4 Nicolò Cusano University (Unicusano), Via Don Carlo Gnocchi 3, I-00166 Rome, Italy; stefano.poponi@unicusano.it; 5 Department of Economics and Law, University of Macerata, Via Armaroli 43, I-62100 Macerata, Italy; luca.salvati@unimc.it; 6 Department of Agricultural Science, University of Sassari, Via De Nicola 9, I-07100 Sassari, Italy; gambella@uniss.it

**Keywords:** disciplinary perspectives, historical narrative, combating desertification, assessment, complex systems

## Abstract

Assuming the importance of a “socioeconomic mosaic” influencing soil and land degradation at the landscape scale, spatial contexts should be considered in the analysis of desertification risk as a base for the design of appropriate counteracting strategies. A holistic approach grounded on a multi-scale qualitative and quantitative assessment is required to identify optimal development strategies regulating the socioeconomic dimensions of land degradation. In the last few decades, the operational thinking at the base of a comprehensive, holistic theory of land degradation evolved toward many different conceptual steps. Moving from empirical, qualitative and unstructured frameworks to a more structured, rational and articulated thinking, such theoretical approaches have been usually oriented toward complex and non-linear dynamics benefiting from progressive and refined approximations. Based on these premises, eleven disciplinary approaches were identified and commented extensively on in the present study, and were classified along a gradient of increasing complexity, from more qualitative and de-structured frameworks to more articulated, non-linear thinking aimed at interpreting the intrinsic fragmentation and heterogeneity of environmental and socioeconomic processes underlying land degradation. Identifying, reviewing and classifying such approaches demonstrated that the evolution of global thinking in land degradation was intimately non-linear, developing narrative and deductive approaches together with inferential, experimentally oriented visions. Focusing specifically on advanced economies in the world, our review contributes to systematize multiple—sometimes entropic—interpretations of desertification processes into a more organized framework, giving value to methodological interplays and specific interpretations of the latent processes underlying land degradation.

## 1. Introduction

Rapid and often unpredictable changes in economic structures have occurred over the past few decades, reflecting (more or less rapid) transitions in societies and local communities, and progressively spreading from affluent to emerging countries. These changes have altered the availability of natural resources and the pristine configuration of rural landscapes, with a (mostly) negative impact on ecosystem functions and services [1,2,3,4]. Structural changes in productive activities, labor markets, housing, and the social organization of life in both advanced and developing countries have determined increasing pressures on fragile ecosystems, especially in the most sensitive and economically disadvantaged areas. These transformations, shaped by a continuous interplay of biophysical and anthropogenic factors, have attracted the increasing attention of social scientists in recent times [5,6,7,8]. While the geography of social inequalities worldwide has been sometimes hypothesized to coincide with climate aridity and poor soils [1,2,6], land degradation seems to be a more subtle phenomenon, affecting both emerging and affluent economies. When political strategies appear to be inadequate to solve environmental problems, under weak institutional and market contexts, the conditions of economic marginality may accelerate land degradation, creating conditions for a vicious spiral toward desertification risk. In these regards, land degradation has been demonstrated to amplify economic polarizations over space, exacerbating social disparities between land degradation free and prone areas [9,10,11,12]. In this line of thinking, desertification can be seen as an extreme form of land degradation in arid, semi-arid and dry sub-humid regions resulting from adverse human impact, in accordance with the traditional definition provided by the United Nations Convention to Combat Desertification (UNCCD).

Scholars and practitioners have been increasingly aware of the drastic consequences of land degradation on socio-demographic and economic systems, evidencing, in many cases, the inappropriate response of local communities to such changes [13]. Uncertainty and risk have been interpreted as key properties of such developmental paths, affecting economic dynamics, social change and political action [14]. Without effective adaptation and mitigation measures facing climate change and responding to individual changes in lifestyles toward more unsustainable models, the “business-as-usual” development path will increasingly promote multifaceted and continuously interacting processes of environmental degradation [15]. Socioeconomic systems are heterogeneous, reflecting past and current disturbance regimes, as well as diverging economic structures and land use. Assuming the importance of a “socioeconomic mosaic” influencing land degradation at the local scale, the differentiated spatial contexts characteristic of local communities—mostly in rural areas—need to be considered in an analysis of land degradation and as the basis for design of appropriate counteracting strategies that face synergistically with biophysical and socioeconomic drivers of change [16,17,18]. A holistic approach based on multi-scalar qualitative and quantitative assessments is thus required to identify the optimal development strategies for the governance of the socioeconomic component of degraded land [19].

Positive and normative debates on the spatial organization of countries and regions are particularly intense in advanced economies, especially where a reduced accessibility of internal, hilly and mountainous areas negatively influences local development, making the relationship between intensive and subsistence agriculture more “spatially asymmetric”, fueling, e.g., rural-to-urban migrations [20,21,22]. At the same time, long-term economic growth has left negative signs on traditional landscapes that were progressively losing the widespread rural traits characteristic of both wealthier countries and emerging economies up to the last decades of the 20th century [23,24,25]. In all of these contexts, rural landscapes started becoming urbanized and served by a progressively more articulated network of infrastructures [26,27,28]. More frequently than expected, both in Europe and in Africa, in North America as well as in Central Asia, urban expansion, industrial development, land-use change and coastalization have affected traditional agro-ecosystems, causing point and diffused soil deterioration [29]. Sprawl, together with crop intensification, industrial livestock, overgrazing and more intense wildfires, has manipulated the typical features of rural landscapes, creating a diversified, and highly fragmented, mosaic of land uses, with coexisting urban and rural settlements [30]. In both advanced and emerging economies, fragmentation of agro-ecosystems leading to land degradation was becoming the new mark of (modern) non-urban landscapes, possibly threatening ecosystem functions and services—the most promising capital endowments for future growth [31,32,33]. In this regard, urban sprawl is becoming a new form of environmental degradation, leveraging desertification risk in advanced economies and, more recently, in some emerging countries with capitalistic accumulation and a rapid increase of income disparities [29,30].

At the same time, marginal districts that have preserved biodiversity for centuries (especially in internal, mountainous regions), are indirectly affected by more subtle forms of dispersed urbanization, fueling rural depopulation and alimenting a downward spiral toward land abandonment [34,35,36]. For instance, in Europe, hyper-rural areas—originally rich at the beginning of the last century—are becoming more and more economically depressed, reflecting increased disparities with flat and more accessible districts being profoundly less rich than one century ago. A sort of “spatial resonance, linking the effects of landscape transformations along the entire urban–rural gradient across regions, countries and continents, is considered to be the latent engine of large-scale mechanisms of land degradation [36]. Considering together causes and consequences of land degradation delineated in earlier sections [37], a global ensemble of socioeconomic syndromes of land degradation based on distinctive mechanisms specifically observed in advanced, emerging and disadvantaged economies was illustrated in Table 1. Syndromes indicate specific processes involving multiple socioeconomic factors under specific ecological conditions, and in turn delineating (more or less evident) environmental consequences. These syndromes were also classified along a temporal gradient from the early 1950s up to now and are assumed to evolve over time, moving from classical factors of land degradation in the immediate aftermath of World War II (e.g., population, agriculture) to more refined processes of change involving tourism, industry, lifestyles, commodities and settlement types. Urban dispersion, international migrations and land grabbing have been considered key drivers of desertification in the most recent decades. As summarized in Table 1, the multiple issues at stake reflect the intrinsic complexity of desertification in all the socioeconomic contexts evaluated in this study. These have frequently resulted in a vast set of theoretical frameworks and empirical approaches to land degradation. Because of their inherent articulation, these frameworks should be characterized and summarized into unique profiles, contributing to an improved comprehension of the (multivariate) mechanisms underlying land degradation.

## 2. The Evolution of Land Degradation Thinking: From Linear Approaches to Circular Economy

Being intrinsically linked to developmental policies combating land degradation, any strategy of sustainable development (e.g., being together economically viable, socially cohesive and environmentally friendly, in line with the three pillars of sustainability) is grounded in the complex interaction between biophysical and socioeconomic factors [37]. However, despite important contributions, nothing definite is said on land degradation and desertification risk, and scientific thinking is still evolving, and delineates innovative approaches to both paradigms [38,39,40]. Operational approaches building a comprehensive “theory” of land degradation evolved toward many different steps, moving from empirical, qualitative and de-structured approaches to more structured notions, usually oriented toward complex and non-linear dynamics based on progressive and refined approximations [41,42,43]. Examples of the inherent shift from linear approaches to complex thinking in the field of land degradation are illustrated and reported in Table 2.

Capturing the intrinsic fragmentation and heterogeneity of environmental and socioeconomic processes that underlie land degradation, 11 disciplinary approaches were identified in this study and classified along a theoretical gradient of increasing complexity, from qualitative (and de-structured) frameworks to more articulated (non-linear) thinking [44,45,46]. The evolution of global thinking in land degradation, however, proved to be intimately non-linear, developing narrative and deductive approaches vis à vis inferential and experimentally oriented visions [47,48,49]. Without ranking the effectiveness and appropriateness of any individual methodology, our commentary is intended as a contribution to systematization and de-complexification of multiple thinking into more organized frameworks based on shared typologies, giving value to methodological interplays and common interpretations of latent processes underlying land degradation [50,51,52].

### 2.1. Historical Narrative

Suggesting investigation approaches that cover long-term changes in natural and human systems [53], land degradation and desertification risk are intriguing issues for historical disciplines perceiving globalization as a recent phenomenon with local interdependencies. Narrative frameworks were usually aimed at exploring basic characteristics and the underlying concepts of land degradation, discussing—likely better than other approaches—the extent to which land degradation constitutes a regional or a global issue, based on the necessary long-term perspective typical of a historical perspective [54]. Frequently, historical approaches have addressed land degradation from a geographical perspective, relating it to broader concepts from both the natural side (such as climate change, drought, and desertification) and the human side (sustainable development). Basically, historical perspectives on land degradation were aimed at showing that, while deserts have been a persistent element of landscapes in human history, dryland degradation is a more recent issue, being primarily associated with the increasing human pressure on the environment [55]. At the same time, the historical approach has clarified how some global issues related to land degradation may reflect global interdependencies that affect several dryland regions worldwide. Narrative approaches definitely pointed out the relevance of land degradation in the ecosystem functioning debate over short time scales (decades), while regarding deserts as a more stable element of global environments over longer time intervals, from centuries to millennia [56]. A refined vision of land degradation and desertification based on the interplay of processes at both time scales is particularly appropriate for environmental governance at large [57] and for future research on globalization and ecosystem quality [58]. With identification, recovery and development of traditional practices being the most relevant targets of this disciplinary approach, ethnographic surveys, visual analysis of present and past landscapes (e.g., using photographs), as well as interviews with privileged witnesses have been the most widely used techniques supporting a historical interpretation of land degradation processes [59].

### 2.2. Controlling Desertification

Landscapes are heterogeneous, reflecting past and current land uses and disturbance regimes as well as different morphology, substrates and ecological mosaics [60]. Assuming the importance of ecological mosaics influencing desertification risk, spatial contexts should be considered in the analysis of land degradation, representing a knowledge base for design of appropriate counteracting strategies [61]. Despite extensive research, a comprehensive classification of the causes of land degradation is still unavailable [62]. While several causes—with both biophysical and socioeconomic origins—have leveraged land degradation, natural causes are probably the most frequently investigated in mainstream literature [63]. Some studies [e.g., 18,35,40] also indicate natural causes of land degradation as exacerbating the severity and impacts of specific anthropogenic activities. Natural causes include, for instance, climate change, whose effects are accelerated by specific land deterioration processes [64]. In this regard, climate change (leading to structural aridity and more intense drought regimes) was frequently seen as the most impactful driver of land degradation, exacerbating soil degradation processes (e.g., erosion, salinization, natural compaction) and accelerating inappropriate (and often unsustainable) human responses, leading to land-use change, landscape transformations and agricultural intensification [65].

Based on these premises, land degradation in the so-called “controlling desertification” approach, is regarded as an integrated biophysical-ecological problem, with desertification risk being driven (or exalted) by climate change [66]. Methodological techniques typical of ecology were adopted in such a disciplinary context, including exploratory data analysis based on multivariate statistics, Geographic Information Systems and Decision Support Systems; remote sensing; field survey of soil, vegetation and water; climate analysis; and the use of simplified biophysical indicators [67]. Together with extensive monitoring, mitigating the effect of (and adapting to) climate change was considered the appropriate response to an increased desertification risk [68]. Landscape restoration is an example of the practical approach typical of the “controlling desertification” vision, also in the face of the debate on (philosophical and operational) differences between rewilding and restoring an ecologically degraded landscape [69]. Socioeconomic issues have been considered only marginally in this perspective, although population density was sometimes regarded as a risk factor [70]. However, population density was uniquely considered from a strictly “ecological” perspective, i.e., as a factor of human pressure per sé, and not for the latent—and likely more relevant—interplay with other socioeconomic dimensions of change.

### 2.3. Ecosystem Services

Ecosystem services are intended as appropriate environmental structures, functions, or processes that directly (or indirectly) contribute to human well-being [71]. Land capital is a particularly useful concept to highlight, measure, and value the degree of dependence between humans and nature [72]. Ecosystem services constitute a powerful tool for assessing ecological status and the sustainability of natural systems [73], and to allow an evaluation of benefits usually excluded in conventional cost-benefit analysis (e.g., environmental, off-site and wider societal benefits). Many of the ecosystem services are the direct result of landscape functioning and De Groot et al. [74] considered their incorporation in landscape analysis and management, with a direct application in the field of land degradation.

In these regards, the notion of “Ecosystem Services” is now progressing in many spheres, analysis dimensions, disciplinary approaches and countries [75]. More specifically, land degradation was intended as a disturbance of an ecosystem’s functioning and biodiversity and, in this regard, monetary evaluations of ecosystem service loss are increasingly required [76]. These estimations may incorporate economic and environmental variables separately or jointly. Mixed approaches integrating biophysical and socioeconomic indicators are revealed to be appropriate when developing interpretative frameworks of land degradation in light of ecosystem services and biodiversity visions [77]. One of the most relevant and practical applications with interest for desertification mitigation includes ecological restoration [78]. However, this notion seems to be less explicitly applied in arid land restoration, a basic issue when referring to ecosystem services. Approaches for quantification and regionalization of Ecosystem Services (ES) has benefited from tools typical of Geographic Information Systems, contributing to an identification of hotspots as a basis for the prioritization of restorative action [79]. Characterizing land degradation effects on ecosystem services may finally benefit from spatially explicit measures of the impact of human consumption or “demand” on environmental functions and services quantified using (direct or indirect) indicators of human appropriation of net primary productivity estimated from official statistics and other appropriate sources [80].

### 2.4. Sustainable Land Management

Combating land degradation (basically at local scale) is increasingly regarded as a challenge for sustainable development strategies [81]. In these regards, the Agenda 2030 redesigned, after extensive negotiation, the objectives and targets of sustainable development, articulating them into objectives and targets, and including the United Nations member states and civil society in the action plan [58]. The resolution of the objectives consists of a broad intergovernmental agreement that constitutes the Post 2015 Development Agenda, aimed at replacing the Millennium Development Goals (MDGs). Unlike the MDGs, the framework of objectives does not distinguish between affluent and emerging countries, since they affect all states without distinction, and this strategy appears to be an implicit contribution to the spatially balanced development issue of the sustainability paradigm. The 17 objectives of the 2030 Agenda (Sustainable Development Goals, SDGs) significantly crosses the perimeter of sustainable development, to place themselves philosophically in a higher and pre-ordered dimension. This refers to the broad and shared theme of lifestyles, work, production and consumption, as well as the reproduction of tangible/intangible resources from the individual scale to the collective and organizational level. In this sense, the United Nations resolution of 2015 expressed, with the adoption of the 2030 Agenda, a clear and timely judgment on the unsustainability of the current development model. The idea that sustainability is only an environmental issue, as has surfaced several times in the past few decades, was definitively overcome and an integrated vision of the different dimensions of development was more recently re-affirmed [30]. The highly innovative nature of the Agenda lies in the fact that all countries are called to contribute to the effort to bring the earth system on a path of sustainable development, without any distinction between developed, emerging and developing countries, even if evidently the problems may vary according to the level of development achieved [58].

Within the SDGs, the sustainable land management issue represents a sort of “continuum” of the specific approaches discussed above, integrating a more (or less) orthodox approach “combating desertification”, with a more holistic vision, shared with both theoretical and empirical frameworks oriented toward ecosystem services, biodiversity conservation and human wellbeing broadly speaking [82]. Multi-scalar assessments mixing qualitative and quantitative approaches were considered appropriate to identify the optimal development strategies for governing environmental and socioeconomic components together [83]. Field surveys, Delphi panels, interviews to privileged witnesses and statistical indicators are examples of the most diffused tools supporting the practical application of sustainable land management principles worldwide [84].

### 2.5. Technological Challenges

Although land degradation primarily threatens the livelihoods of rural poor, this interaction is complex and conditioned by socioeconomic and environmental drivers, reflecting the intrinsic, joint benefits of local development and policies combating poverty [85]. In this context, investments that improve the livelihoods of affected societies have been often seen as a comprehensive development strategy, containing outmigration and depopulation in severely impacted areas [86]. Implementation of technical strategies more oriented toward green economy principles leads currently to a progressive spatial re-arrangement of production activity, and agriculture is not an exception to this (more or less) general rule [87]. While—in a pure, Malthusian perspective—land resource scarcity resulting from population growth has caused land-use conflicts up to recent past, degradation narratives may themselves cause aliment in present and future conflicts, legitimizing the way for agricultural investments (e.g., land grabbing) [88]. At the same time, environmental conservation measures under a “purely green” economy seems to be more effective than traditional strategies (like those illustrated above) because of the joint leverage of technology and incentives to green productions [89].

In the backdrop of response policies to operationalize an inclusive green economy indirectly fighting desertification, economic effectiveness of ecosystem restoration is one key analysis dimension [90]. By suggesting that the efficacy of investment restoring land capital is particularly high, metrics to assess an inclusive green economy and the contribution to fight against desertification are increasingly needed, valuing man-made capital, human capital, and land capital as the preferred choice [91]. Alternative options for the economic evaluation of land degradation and actions to combat desertification risk have made available in the literature, including Cost-Benefit Analysis (CBA), Cost–Effectiveness Analysis (CEA) and Multi-Criteria Analysis (MCA). The first approach provides a full monetary valuation of costs and benefits of interventions; the second technique compares costs with a predefined objective of the actions, thus identifying the less expensive alternative. The third procedure provides a broad set of methods aimed at supporting the identification of preferred solutions within a defined set of alternatives evaluated with regard to a predefined list of evaluation criteria or objectives.

Technological challenges, and more specific “green economy” strategies definitely hold the potential to inform policymakers on investment trade-offs between different life domains and distributional arrangements at the level of regional economic systems [92]. Coordination between measures and the appropriate subsidy regime are additional aspects assuring the inherent functioning of such strategies at the local scale [93].

### 2.6. The Traditional “Demand–Supply” Economic Approach

Modeling has been often considered the most appropriate way to investigate feedback interactions between economic systems and land degradation [94]. Microeconomic models seek to explain how individuals allocate their resources, using standard economic variables such as background and preferences, prices, institutions, access to infrastructure and services, as well as technological alternatives [95]. A major distinction could be made between models that assume prices are market-determined and that farmers are fully integrated into perfect markets and those that do not [96]. Within the former category, production decisions are determined by the level of market prices and can be interpreted as a profit-maximizing problem [97]. When farmers are not fully market-integrated, decisions are based on farmers’ subjective (and endogenously determined) shadow prices [98]. Factors such as resource endowments and household composition are important, and the consumption side should be considered when making the production decision [99]. This distinction turns out to be critical for those models that predict soil degradation, since consumption and degradation will vary in response to population growth, changes in agricultural prices, and per-capita average income [100].

National and multi-country (macroeconomic) models emphasize the relationship among underlying variables, decision targets, and the environment [101]. Analytical, simulation, and regression models are suitable at this level [102]. To model complex macroeconomic processes in a strictly analytical framework and achieve interesting conclusions, model makers have had to place limits on the number of variables and make some (strong) assumptions [103]. Both analytical and simulation models developed at the country (or supra-national) level add important analysis dimensions that are absent in household- and firm-level models [104]. First, they make some prices endogenous. By this way, they move beyond simply asking how decision parameters influence agents and look at how the underlying variables determine one particular set of decision parameters [105]. This further provides an important link to macroeconomic variables and policy instruments [106]. Second, most models include the interactions among different sectors, e.g., sub-sectors of agriculture, manufacturing, and services, which makes them useful in the in-depth analysis of factors underlying land degradation [107].

Results of formal models have rarely assured a systematically higher accurateness than results from exploratory analysis grounded on mixed quali-quantitative approaches [108]. Model’s misspecification derives from mixing the different mechanism levels. This reveals that exploratory investigation (e.g., based on quali-quantitative surveys) is, at least in many cases, necessary to clarify causal relations and latent associations among environmental-economic variables [109]. Exploratory investigation of the multifaceted economic factors underlying land degradation (e.g., integrating statistical indicators, remote sensing, field and photographic surveys, interviews to local stakeholders, and archive/bibliographic reviews), was demonstrated to effectively complement formal models in most studies [110].

### 2.7. Political Ecology

Human pressure on ecosystems frequently manifests through unsustainable exploitation of land resources, altering the ecological balance and leading to the progressive depletion of natural capital—sometimes replaced with more accessible economic capital [111]. Forms of human pressure include intensive agriculture, overgrazing, wildfires, mining, urbanization, industrialization, infrastructural development, and seasonal tourist concentration, among others [112]. Within this complex interplay of socioeconomic forces, undermining the role of institutions that regulate (and prevent misuse) of land resources is a necessary step ahead in land degradation policy [113]. Differing from (apolitical) environmental studies by politicizing ecological problems, political ecology offers an interpretative platform to these dynamics, clarifying the intimate relationship of the environmental sphere with multiple political, demographic, cultural, institutional, economic, and social factors [114]. By integrating ecology with political economy, this discipline reframed topics such as soil degradation and farm marginalization, ecological conflicts, conservation, and control of land resources, as well as environmental identities and social movements [115]. Interviews, policy analysis, and statistical indicators are relevant tools providing the empirical knowledge to political ecology theories and assumptions [116].

### 2.8. Environmental Economic Geography

Although with distinctive disciplinary orientation, environmental dimension and sustainability-related issues have increasingly gained momentum in economic geography. Integrating a “spatial inequality” perspective and a “global ecological challenge” vision into basic frameworks oriented toward an Environmental Economic Geography, contributes to disentangle the manifold relationship between socioeconomic and environmental disadvantages [117]. While social inequalities worldwide often correlate over space with climate aridity [58], land degradation seems to be a more subtle process, affecting both emerging and affluent economies [118]. When the political strategies appear inadequate to solve environmental problems and market and/or institutions are weak, poverty and economic disadvantage are demonstrated to accelerate land degradation and vice versa [119]. Poverty, understood as a generalized condition affecting fragile populations, can provide an interpretative key to explain international migration and the abandonment of rural land and/or demographic concentration in urban areas, e.g., reinforcing land degradation in peri-urban districts [120].

When causal mechanisms are not entirely clear and linear because of the latent interplay of many complex factors, approaches oriented toward Environmental Economic Geography thinking may inform governance and market regulation strategies [121]. Strategies aimed at containing socioeconomic imbalances seem definitely to be the most appropriate to guarantee a sustainable development fully integrated with a biophysical vision embedded in a “working with nature” thinking [122]. Smoothing out these imbalances requires effective measures linking the traditional aim of socioeconomic cohesion with relatively new targets of environmental justice [123]. In this ambit, research on the convergence/divergence path of socioeconomic systems exposed to land degradation provides key knowledge informing sustainable land management and spatial planning oriented toward a more balanced local development [124]. At the same time, policies to combat desertification differentiated for regional specificities—not necessarily following administrative boundaries—are in line with a truly environmental economic geography thinking [125].

### 2.9. Complex Adaptive System Thinking

Systems’ analysis indicates feedbacks and its possible effect on any action or restoration practice as a key target of measurement in any land degradation theory [126]. Feedbacks—and especially negative feedbacks—are regarded as part of an equilibrium system and their operation brings systems back to a less disturbed state. At the same time, positive feedbacks lead to increasing instability and non-linear system change, being crucial in relevant processes of land degradation, including accelerated soil erosion and desertification risk [127]. A mostly related notion is that of thresholds typical of the given system, whose crossing may lead to a significant change in a system’s properties or general behavior [128]. By concluding the circle, “slow” or “fast” (according to the temporal scale they interact with), variables accelerate or slow down land degradation, leading to different equilibria of the system [129]. Considering such definitions, the conceptual frameworks proposed for analyzing complex arid and semi-arid landscapes provide basic examples of complex adaptive systems’ functioning. These incorporate and classify feedbacks, thresholds and properties into non-linear interactions amongst (i) historical legacies, (ii) spatial contexts and patterns in relevant ecological variables, (iii) transport processes, (iv) resource redistribution between areas with high and low land stocks, and (vi) feedbacks among plants, animals, and soils [130]. Taken together, these approaches have the merit to clarify the intrinsic linkage between land degradation and the socioeconomic resilience of local systems, a particularly real issue [131]. With this perspective in mind, panarchy, system-level properties, and fast (or slow) drivers of change need to be identified and analyzed in any given context, mainly using quali-quantitative methodologies based on the integration of different techniques and approaches [132].

### 2.10. Land Degradation Neutrality

An integrated reading of land vulnerability levels and their evolution over time provides a dynamic picture of the environmental background conditions leading to a higher risk of desertification [133]. Results of long-term approaches have outlined an evident trend towards the progressive worsening of ecological conditions worldwide [134]. Such processes of land deterioration present a somewhat complex spatial distribution, which often does not allow for the identification of clear geographical gradients [135]. For instance, the level of land vulnerability at the local scale seems to display more structured trends, requiring accurate assessment and interpretation [136]. Results of spatial analysis recommend further consideration of the spread (or concentration) of areas with intrinsic issues of land vulnerability to degradation, revealing the increasing need of effective mitigation policies targeting a zero net land degradation, i.e., a target of a stable (or reduced) level of land degradation over a defined time interval [137]. While being a truly operational and new approach, zero-net land degradation represents a paradigmatic change of perspective, involving all affected territories and avoiding artificial distinctions between “rural” districts and other land [138]. This paradigm clearly focuses on the rapid transition of land vulnerability, increasing sharply in many different places with distinctive—and mostly non-rural characters—that need effective containment policies in addition to mitigation and adaptation strategies [139,140,141].

A practical intervention opportunity came from the REDD strategy, under the assumption that dryland occupies 41% of the earth’s land area and effectively protects the soil from desertification, providing in turn ecosystem services. REDD (Reducing Emissions from Deforestation and forest Degradation) is an international mechanism promoted by UNCCD and aimed at providing developing countries with incentives for the protection and better management of forests in order to avoid emissions from deforestation and forest degradation [142,143,144]. The concept of REDD was introduced to capture the co-benefits of REDD activities by including the roles of biodiversity conservation and sustainable management of forests. In addition to climate change mitigation, REDD in drylands was aimed at (indirectly) alleviating poverty and improving the living conditions of indigenous and local communities by promoting sustainable land management [145,146,147]. Soil conservation, agroforestry and silvo-pastoral practices along with sustainable soil and water management are some of the proven methods for enhancing the ecological and socioeconomic benefits from REDD initiatives in dry areas.

### 2.11. Circular Economy

While economic growth has been demonstrated to produce a negative trade-off with the environment, development paths may stimulate, in most cases, the required contrast/mitigation response to land degradation [139]. However, land degradation does not present a common profile neither on the continental or the national scale, being intimately shaped by different productive, institutional, and cultural values [39]. These findings prevent a complete understanding of the environmental mechanisms beyond resource degradation, which differ significantly across regions and countries [46]. The transmission channels of socioeconomic impacts on the ecosystem also present some intrinsic characteristics that make it difficult to apply a unique interpretative paradigm to investigate place-based specificities referring to such processes [98]. With this perspective in mind, the circular economy is a modern kind of thinking that may adapt more rapidly to such issues, since it provides a holistic and flexible vision of the ecological–economic nexus at the same time [63]. While improving the intrinsic productivity of capitals, circular systems employ reuse, sharing, repair, refurbishment, remanufacturing, and recycling to create a close-loop system [138]. This rationale represents (more or less) the opposite thinking that characterizes land degradation [148]: minimizing the use of resource inputs and the creation of emissions is likely the most effective (while indirect) response to the unsustainability path delineated by land degradation [13]. Regenerative resources contrast with traditionally linear economic systems, which have a “take, make, dispose” model of production at the base of land degradation [73]. Being achieved without a significant loss of revenue, circular economy thinking testifies how a sustainable world includes profitable (circular) business models when the appropriate production chain—having null (or negligible) impacts on land—is established [74]. Future research should focus on operational frameworks linking circular economy thinking to land degradation mitigation, basing on the causal relationship between individual production chains and specific processes of soil deterioration on the same spatial and organizational scales [139].

## 3. Concluding Remarks

While in light of projected climate change and increased human pressure, traditional approaches may not be feasible (or economic) to assess land degradation, de-complexifying theories and empirics into specific disciplinary (or paradigmatic) profiles were assumed to be effective in grasping the intimate articulation of this issue and the required monitoring strategies [113]. In the present study, 11 paradigmatic visions were proposed to identify and classify a number of studies addressing land degradation into unifying disciplinary profiles, giving value to multi-scalar, multi-temporal, multi-sectoral, and multi-disciplinary approaches. The increased relevance of the “land degradation” notion in recent debates on climate change, human pressure, food security, and public health—just to mention the most relevant and actual topics—definitely confirms the need for a comprehensive approach to sustainable development [37]. Expected to represent an equally balanced growth path across time, space, and productive sectors, sustainable development definitely links separate issues in a common, paradigmatic vision of the future global path of economies, societies, and environments, assuring safe conditions for life on the earth, ecologically functioning and bio-diverse ecosystems, and economically viable systems with cohesive and equal societies.

According to Blaikie and Brookfield [3], land degradation (and desertification risk in the broad sense) were taken as exquisitely socioeconomic problems, since land exploitation and management derive from social needs. Soil productivity, the bearing capacity of ecosystems, conflicts for access to natural resources and, ultimately, the pursuit of sustainable development, are the intrinsic products of dynamic human-nature interactions [44]. With this framework in mind, reducing territorial disparities between urban and rural areas, coastal and inland districts, rich and poor regions, is clearly a strategic way of sustainable development combating land degradation and reducing the intrinsic risk of desertification, especially in arid contexts. This assumption highlights the importance of the spatial organization of regions and countries, the integration of economic contexts, and the reduction of social inequalities, all being reflected in the inherent transition from linear approaches to a more complex thinking [36], as reflected in the present study. The literature scrutinized in this paper and, consequently, the typological approaches identified and profiled, represent a sort of operational gradient from a linear, simplified, and mostly one-dimensional interpretation of land degradation, to a more complex, non-linear and articulated approaches to multi-dimensional local systems experiencing a progressive transition toward sustainable (or unsustainable) development [148,149,150].

An extensive review of theories underlying land degradation outlines a rethinking of future research focusing specifically on the role of technological change, the impact of credit markets, the mitigation of demographic conflicts for environmental resources, the safe provision of food/fiber and healthy conditions of life, and the quantification of socioeconomic resilience of local systems [25]. In the long term, it should be necessary to integrate more tightly the (mostly uni-dimensional) studies focusing on land degradation within the mainstream literature dealing with global change [151], downscaling the scenarios available at the continental scale up to sub-national contexts, in order to clarify the main drivers of change in local socio-ecosystems [40]. From the economic side, systematic studies should be promoted on the costs and effectiveness of the various strategies available at the country level, in connection with local case studies comparing alternative evaluation techniques [10]. Practical demonstrations, pilot studies and research on local communities in which the application of alternative strategies is explored, could support the implementation of adaptive approaches towards combating land degradation more in line with the taxonomy proposed in this commentary [85,142,143]. Applying the proposed taxonomy when designing future (empirical) studies at the local scale seems to be a practical solution, giving value to both positive and normative aspects of any investigation in the field of land degradation.

Being consistent with the concept of sustainable development, effective approaches to land degradation complexity should overcome a narrow sectoral vision in order to pursue a truly multi-scalar approach [19]. This notion is especially important when different degradation processes act synergistically, displaying interactions and feedbacks that are hardly coordinated and regulated on a local scale [15]. While the “local” dimension remains a fundamental prerequisite for both theoretical approaches and intervention strategies, land degradation claims for more coordinated sustainability strategies conceived on a national or supranational scale with the aim at mitigating spatial imbalances [69]. The notion of “sustainable development”—understood as a spatially balanced process in all the composing dimensions over both time and space [151]—makes a useful contribution to this knowledge path. This assumption is particularly appropriate not only because the “sustainability” notion is independent of the specific definition of development. What is especially important here is that the identification of policy and feedback objectives and mechanisms that incorporate sustainability actions and best practices for land degradation mitigation may adapt to a changing (e.g., rising) risk of desertification [4]. In these regards, the probability that a truly sustainable development path will materialize in economic policy measures is complex to quantify and predict, being undoubtedly linked to the dynamic relationship between the time horizon of policy makers and the effects of non-sustainability.

## Figures and Tables

**Table 1:** Exploring the desertification–economic nexus: an example of a global downward spiral leading to land degradation since World War II distinguishing specific syndromes in different world regions.

Region	1950–2020				
Affluent countries	Population growth and late industrialization	Crop intensification; “baby boom”	Tourism development; globalization; metropolization	Social change toward unsustainable life styles	Urban sprawl and intense land take in rural areas
Rapidly emerging countries	Land abandonment in less accessible rural areas	Agricultural revolution and massive land-use change altering regional landscapes; demographic transition		Drastic population disparities along urban–rural gradients; tourism development in ecologically fragile districts	
Disadvantaged, late-development countries	Population shrinkage; rural marginalization inland	Urban expansion with (or without) industrial development; intense demographic growth		Migration to developed regions	Land grabbing

**Table 2:** The evolution of socioeconomic thinking of land degradation, distinguishing approaches mainly oriented toward Research (R), Practical (individual) actions (A), Policy and governance (P).

Philosophy/Approach	Brief Description/Key Words	Methodologies	Application	Time *
Historical narrative	Identification, recovery and development of traditional practices	Ethnographic survey, visual analysis of past landscapes, interviews with privileged witnesses	R/A	1970s–1980s
“Controlling desertification”, extensive monitoring, landscape restoration, mitigation/adaptation to climate change	Land degradation as an integrated biophysical-ecological problem; desertification as a process driven or exalted by climate change; socioeconomic issues considered only marginally; population density occasionally considered as a risk factor	Remote sensing, field survey (soil, vegetation), climate analysis; other biophysical indicators; past and actual landscape photographs; multivariate statistics	R/P	1970s
Ecosystem services/biodiversity perspective	Land degradation as a disturbance of an ecosystem’s functioning and biodiversity; monetary evaluation of ecosystem service loss; Millennium Ecosystem Assessment	Geographic information systems, biophysical indicators; socioeconomic indicators	R/P	1980s
Sustainable land management	Combating land degradation (basically at the local scale) as a challenge for sustainable development strategies	Field surveys, Delphi panels, interviews, Agenda 21 indicators	A/P	1990s
Technological challenges	Technological solutions to desertification (e.g., restoration); traditional engineering approaches and green economy options	Life cycle assessment	A	1980s
The traditional demand–supply approach	Economic theory, equilibrium simulations, data-driven exercises	Econometric models (e.g., panel regressions)	R/P	Late 1980s
Political ecology	Understanding the role of institutions regulating use and preventing misuse of land resources	Interviews, policy analysis, statistical indicators	R/P	1990s
Environmental economic geography	Socioeconomic disparities as the engine of land degradation at country/continental scale; territorial cohesion as a pillar of sustainability strategies; spatial justice; combating territorial imbalances as a strategy to fight desertification risk	Statistical indicators, Spatial analysis	R/P	Late 1990s
Complex adaptive system thinking	Land degradation and socioeconomic resilience; panarchy; system-level properties; fast-slow drivers of change	Quali-quantitative holistic approaches	R/P	Early 2000s
Land degradation neutrality	Policy-oriented targets following Sustainable Development Goals	Indicator dashboards	P	Late 2000s
Circular economy	Full integration of land degradation issues in the economic system, integrating past knowledge with new technical solutions	Mixed quali-quantitative approach	R	Early 2010s

* estimates the time interval with the first (and likely most relevant) contributions in the field, based on a subjective analysis of literature.
